# Handwashing and Ebola virus disease outbreaks: A randomized comparison of soap, hand sanitizer, and 0.05% chlorine solutions on the inactivation and removal of model organisms Phi6 and *E*. *coli* from hands and persistence in rinse water

**DOI:** 10.1371/journal.pone.0172734

**Published:** 2017-02-23

**Authors:** Marlene K. Wolfe, Karin Gallandat, Kyle Daniels, Anne Marie Desmarais, Pamela Scheinman, Daniele Lantagne

**Affiliations:** 1 Department of Civil and Environmental Engineering, Tufts University, Medford, Massachusetts, United States of America; 2 Department of Dermatology, Brigham and Women’s Hospital, Boston, Massachusetts, United States of America; University of Ottawa, CANADA

## Abstract

To prevent Ebola transmission, frequent handwashing is recommended in Ebola Treatment Units and communities. However, little is known about which handwashing protocol is most efficacious. We evaluated six handwashing protocols (soap and water, alcohol-based hand sanitizer (ABHS), and 0.05% sodium dichloroisocyanurate, high-test hypochlorite, and stabilized and non-stabilized sodium hypochlorite solutions) for 1) efficacy of handwashing on the removal and inactivation of non-pathogenic model organisms and, 2) persistence of organisms in rinse water. Model organisms *E*. *coli* and bacteriophage Phi6 were used to evaluate handwashing with and without organic load added to simulate bodily fluids. Hands were inoculated with test organisms, washed, and rinsed using a glove juice method to retrieve remaining organisms. Impact was estimated by comparing the log reduction in organisms after handwashing to the log reduction without handwashing. Rinse water was collected to test for persistence of organisms. Handwashing resulted in a 1.94–3.01 log reduction in *E*. *coli* concentration without, and 2.18–3.34 with, soil load; and a 2.44–3.06 log reduction in Phi6 without, and 2.71–3.69 with, soil load. HTH performed most consistently well, with significantly greater log reductions than other handwashing protocols in three models. However, the magnitude of handwashing efficacy differences was small, suggesting protocols are similarly efficacious. Rinse water demonstrated a 0.28–4.77 log reduction in remaining *E*. *coli* without, and 0.21–4.49 with, soil load and a 1.26–2.02 log reduction in Phi6 without, and 1.30–2.20 with, soil load. Chlorine resulted in significantly less persistence of *E*. *coli* in both conditions and Phi6 without soil load in rinse water (p<0.001). Thus, chlorine-based methods may offer a benefit of reducing persistence in rinse water. We recommend responders use the most practical handwashing method to ensure hand hygiene in Ebola contexts, considering the potential benefit of chlorine-based methods in rinse water persistence.

## Introduction

The Ebola Virus Disease (EVD) outbreak in West Africa from 2013–2016 was the largest to date, resulting in 28,638 cases, 11,316 deaths, and establishing the disease as endemic in the region [1]. From the time the Ebola virus was first identified in 1977 until the outbreak in West Africa the disease occurred in rural areas and its spread was limited by geography and rapid medical response [2,3]. The West African outbreak was the first time EVD spread widely through person-to-person transmission, reaching ten countries on three continents [1,4–6].

Ebola is an enveloped, non-segmented, negative sense, single-stranded RNA filovirus with seven genes. The filamentous particles are approximately 80nm in diameter and range from 300–1,100 nm long [7,8]. The virus infects a broad range of cell types, and symptoms begin abruptly, including a high fever, headache, muscle pain, weakness, diarrhea, and vomiting. Many patients experience a characteristic rash and some hemorrhage [5,9]. EVD has a high case fatality rate (ranging from 25–100%) and supportive therapy is the only treatment currently available [6,10].

EVD is spread through contact with infected individuals, their bodily fluids, or contaminated surfaces such as hands [11]. Contact can be difficult to avoid, as people with Ebola produce a large volume of infectious vomit and diarrhea, and sometimes hemorrhage blood which may contain a viral load up to 10^8^/mL [12,13]. Within Ebola Treatment Units (ETUs), workers wear personal protective equipment (PPE) so that no skin is exposed and rigorous standards of handwashing are upheld [14]. For Ebola outbreaks, the most commonly recommended handwashing agents are soap and water, alcohol-based hand sanitizer (ABHS), and 0.05% chlorine solutions [15–18], although recommendations vary by international organization. Doctors Without Borders (MSF) recommends the use of 0.05% chlorine for handwashing during Ebola outbreaks, and buckets of solution are placed around ETUs to allow for regular handwashing [15]. The World Health Organization (WHO) recommends handwashing with ABHS if hands are not visibly soiled and with soap and water (HWWS) for soiled hands, and states that chlorine should be used for handwashing only if other options are not available, because chlorine 1) would be less effective than other methods due to chlorine demand from skin and, 2) could perturb the protective skin barrier and place users at higher risk of Ebola transmission [16–19]. In the West African outbreak, unprecedented person-to-person transmission meant that handwashing recommendations intended for ETUs were extended to homes and public places in communities at risk for EVD. Handwashing with 0.05% chlorine solution was commonly adopted by government, health, and commercial facilities. However, the safety, efficacy, and practicality of these recommendations both in general, and specifically for an EVD outbreak, was unknown.

The purpose of handwashing is to remove potentially harmful organisms from hands, preventing their transmission from person to person. Handwashing may inactivate organisms, but this is not always necessary to prevent disease transmission. Inactivation is not expected for handwashing protocols using plain soap, but is expected for protocols using chlorine. Although handwashing is widely considered to be an important part of disease prevention and studies show that it is effective for preventing transmission of infection [20–23], studies on handwashing efficacy for the removal or inactivation of a range of organisms from hands using different protocols have shown inconsistent results. In an informal literature search, we identified 14 studies that compared the efficacy of HWWS and ABHS on the removal of organisms: of these, seven found HWWS to be more efficacious [24–30], five found ABHS to be more efficacious [31–35], and two found little difference between the methods [36,37]. One of these manuscripts showed that while some soaps were more efficacious than ABHS, others were not. Relatedly, recently the United States Food and Drug Administration banned a suite of chemicals used in antibacterial soaps, stating that there is no evidence that these additives increase the efficacy of handwashing [38]. Lastly, in some cases, it has been shown that the mechanical action of handwashing alone may account for most organism removal [39,40].

A recent systematic review on evidence for handwashing with chlorine in Ebola outbreaks found only four studies investigating the efficacy of chlorine for handwashing [41], and we identified one additional study. None of the five studies were relevant for viruses like Ebola; all were of variable quality and produced conflicting results, and none used the concentration of 0.05% that is internationally recommended for handwashing [25,42–46]. Overall, the current evidence about efficacy of different handwashing protocols in Ebola outbreaks is limited and of low quality and we did not identify any studies evaluating the persistence of organisms in rinse water.

Along with safety and efficacy considerations, there are benefits and drawbacks to each handwashing protocol in outbreak settings, including ease of transport, local availability, cost, and acceptability (Table 1). Soap is used for handwashing worldwide and is generally available, familiar, and acceptable in communities that have experienced Ebola outbreaks. However, soap requires water for use, and bars of soap can be easily lost or stolen from handwashing stations. ABHS does not require water and is easy to use, however it is usually imported, costly, and is sometimes prohibited by Muslim communities in West Africa who may consider use to be consumption of alcohol [20]. The WHO does provide guidance for ABHS formulas that can be produced locally for a lower cost than imported products, however the cost of materials is variable and significant time and resources are needed to ensure high-quality production [47]. Chlorine solutions are widely used and accepted for handwashing in Ebola contexts [15]. However, producing high-quality chlorine solutions requires accurate measuring instruments, testing methods to confirm the concentration of solutions [48], and dilution water with no chlorine demand. Additionally, there are four chlorine compounds used in outbreak response: high-test hypochlorite (HTH), locally-generated and stabilized sodium hypochlorite (NaOCl), and sodium dichloroisocyanurate (NaDCC). Each chlorine type has benefits and drawbacks, as detailed in Table 1. Due to the relative lack of drawbacks, NaDCC has become commonly used in ETUs.

**Table 1:** Benefits and drawbacks of commonly used handwashing protocols.

Handwashing Protocol	Benefits	Drawbacks
**Soap and Water**	Widely available, acceptable	Does not inactivate pathogens, requires water
**Alcohol-Based Hand Sanitizer**	Simple, portable	Not widely acceptable or available, expensive
**NaDCC** (pH = 6)	Easy to ship (powdered), long shelf-life, does not clog pipes	
**HTH** (pH = 11)	Easy to ship (powdered), long shelf-life	Can cause explosions, clogs pipes
**NaOCl** (pH = 11)	Can be locally produced, does not clog pipes	Shorter shelf-life, difficult to ship
**Generated NaOCl** (pH = 9–11)	Can be produced on-site, does not clog pipes	Shorter shelf-life, difficult to ship, quality control/manufacturing

Benefits and drawbacks reflect the expert opinion of responders working with these handwashing protocols during emergency response.

The selection of a safe, efficacious, and practical handwashing protocol is essential during an EVD outbreak, and is currently hampered by limited evidence on the efficacy of commonly used handwashing protocols and lack of evidence about persistence in rinse water. To address these research gaps, we evaluated six commonly recommended handwashing protocols for: 1) efficacy at the removal of non-pathogenic model organisms from hands and, 2) ability to reduce persistence of these organisms in rinse water.

## Methods

We conducted testing with human subjects to investigate the reduction of non-pathogenic model organisms on hands after washing and the persistence of organisms in rinse water using a matrix including six handwashing protocols and two controls, two surrogate organisms, and two soil load conditions. This was a crossover randomized trial in which surrogate organisms and soil load status were tested on the same subjects at different times, and in which the order of application of the handwashing protocols was randomized for each subject at each time. The study enrollment and baseline, materials used, and experimental procedure are described below.

### Enrollment and baseline

The study took place on the Tufts University campus in Medford, MA, USA beginning with recruitment on April 2, 2016 and concluding with the final experiments on May 5, 2016. The study was approved by the Institutional Review Board at Tufts Medical Center and Tufts University Health Sciences Campus (#12018); Harvard University ceded review to the Tufts Institutional Review Board.

A total of 18 volunteers were recruited and signed written informed consent forms. This sample size was selected based on the average size of similar efficacy studies, and to allow for standard statistical tests [24,25,36,39,49]. Volunteers were required to be healthy, between the ages of 18 and 65, and with no skin damage or disorders, known allergies to the handwashing materials, or history of mental health issues related to hygiene practices. Volunteers were unable to participate if they were currently taking antibiotics or pregnant. After enrollment, volunteers responded to a brief baseline questionnaire, including demographic information, personal history of skin conditions, and information about recent handwashing behavior. A researcher trained by a board-certified dermatologist then conducted a visual hand examination to look for signs of current dermatitis, hand injuries, or baseline skin abnormalities. Volunteers were instructed to avoid antimicrobial products for the seven days prior to each test and were provided with donated Free and Clear Shampoo and Conditioner and a Vanicream^™^ Cleansing Bar (Pharmaceutical Specialties, Inc. Rochester, MN) to use in place of their usual products. Commercially available heavy-duty vinyl gloves (Allerderm, Phoenix, AZ) were also provided for cases in which contact with antimicrobial products was unavoidable (e.g. dishwashing).

### Materials preparation

#### Handwashing solutions

The six handwashing protocols were HWWS, ABHS, 0.05% HTH solution, 0.05% NaDCC solution, 0.05% NaOCl solution generated with an electrochlorinator, and stabilized 0.05% NaOCl solution produced from laboratory-grade NaOCl stock. These were compared to two controls: no handwashing (control A) and handwashing with water only (control B).

For HWWS, commercially-available Dove White Beauty Bar soap (Unilever, Trumbull, CT) was used. For ABHS, Purell Advanced Instant Hand Sanitizer with 70% Ethyl Alcohol (GOJO Industries, Inc. Akron, OH) was used. Chlorine solutions were produced at the Environmental Sustainability Laboratory at Tufts University. Generated NaOCl was produced with laboratory-grade sodium chloride and MilliQ water using an AquaChlor on-site sodium hypochlorite generator (International Equipment & Systems, Inc. Miami, FL). Stabilized NaOCl was produced by diluting a 5.25% laboratory-grade pH stabilized bleach stock solution (Valtech, Zellenople, PA) with MilliQ water. NaDCC was produced using Klorsept (previously Aquatabs) granules with 50% available chlorine (Medentech, Wexford, Ireland) in MilliQ water. HTH was produced using commercially available granular calcium hypochlorite with 65% available chlorine (Acros Organics, New Jersey, USA) in MilliQ water. Concentrations were confirmed to be within 10% of the goal concentration of 0.05% on the day of the experiment using Hach Iodometric Titration Method 8209 (Hach Company, Loveland, CO).

#### Organisms

To extend the relevance of our work beyond Ebola and into other possible organisms of interest, we used non-pathogenic *E*. *coli* as a bacterial comparison and potentially relevant indicator for other diseases of public health importance. *E*. *coli* has been commonly used as an indicator for hand hygiene studies, and is recommended in the ASTM International Methods [39,50]. In the week prior to experiments, we streaked a nonpathogenic strain of *E*. *coli* (ATCC^®^ 25922) onto Luria-Bertani (LB) agar plates and incubated at 35°C for 24 hours to obtain singles colonies before storing at 4°C. The evening prior to an experiment, a single colony from the plate was used to inoculate 10mL of LB broth and incubated overnight at 35°C with shaking. On the morning of the experiment, a fresh culture was launched with 1mL of overnight culture in 20mL fresh broth and incubated for approximately 2.5 hours. A GeneQuant^™^ 100 spectrophotometer (GE Healthcare, Marlborough, MA) was used to estimate the concentration of the culture for a target of greater than 10^8^ CFU/mL. This culture was then used to make an inoculate composed of 68% culture and 32% soil load or 0.9% NaCl solution (depending on the condition tested) to spike hands. Concentration of inoculate was confirmed using membrane filtration; samples were passed through a 0.45 μm filter (EMD Millipore, Billerica, MA) and incubated on m-ColiBlue24 media (Hach Company, Loveland, CO) for 24 hours at 35°C. Approximately 20mL of phosphate-buffered saline (PBS) was added to the filtration funnel prior to filtering any samples less than 10mL in volume to ensure uniform filtration of the sample.

We chose Phi6 as a non-pathogenic, biosafety-level 1 bacteriophage surrogate for the biosafety-level 4 Ebola virus (which ethically cannot be placed on human hands) for this experiment. Phi6, like Ebola, is enveloped, and has previously been used as a surrogate for enveloped viruses in environmental studies and has previously been used in studies on human hands [51–54]. As part of the larger project this work was completed with our group completed a study to compare several potential BSL-1 surrogate organisms (including MS2, PR772, Phi6, and M13) to published data on efficacy of surface disinfection with chlorine against the actual Ebola virus [46,55]. We found that Phi6 was slightly more resistant to chlorine on surfaces than the Ebola virus, and as such selected Phi6 as a slightly conservative surface disinfection indicator for the Ebola virus.

The Phi6 bacteriophage (HER #102) was propagated in the *Pseudomonas syringae* (HER #1102) host by the double agar overlay method [56]. Both 100μL Phi6 stock suspension and 100μL *P*. *syringae* overnight culture were pipetted directly into 6mL of Nutrient Broth Yeast (NBY) soft agar (0.3%) and poured onto plates with NBY hard agar (1.5%). Plates were incubated at 26°C for 24 hours. Phi6 was retrieved by diffusion [57]; 5mL of phosphate buffered saline (PBS) was added on top of the soft agar layer, left at room temperature for four hours, then retrieved with a pipette, filtered at 0.45μm, and stored at 4°C. The stock was used to make an inoculate composed of 68% viral stock and 32% soil load or PBS (depending on the condition tested) on the day of the experiment to spike hands. Plaque assay was used to confirm a concentration greater than 10^7^ PFU/mL; 100 μL of diluted sample was pipetted directly into 6mL of NBY soft agar with 100 μL of overnight host culture, poured over NBY hard agar, and incubated at 26°C for 24 hours [56].

#### Soil load

Because EVD patients generally shed virus within bodily fluids such as blood, diarrhea, and vomit, and these fluids may alter handwashing efficacy, each condition was also tested with and without added soil load mimicking human bodily fluids. A tripartite soil load was prepared based on ASTM International Standards, containing 7.80 mg/mL bovine serum albumin, 10.92 mg/mL tryptone, and 2.52 mg/mL bovine mucin and added to *E*. *coli* and Phi6 inoculates [58].

### Experimental protocol

Volunteers were asked to come to the Environmental Sustainability Laboratory at Tufts University for testing of four conditions: 1) *E*. *coli* without soil load; 2) *E*. *coli* with soil load; 3) Phi6 without soil load; and, 4) Phi6 with soil load. Each condition was administered on a separate day. Before taking samples each day, researchers confirmed eligiblity and fitness. First, volunteers responded to a short survey about their adherence to the antimicrobial-avoidance period and received a visual hand exam to confirm the absence of breaks or abnormalities in the skin. If lack of adherence or skin breakage was noted, volunteers were unable to participate in that day’s experiment. Second, each volunteer was randomly assigned the left or right hand for the day’s experiment and an order in which the six handwashing protocols and two controls (eight conditions total) would be tested. Third, a “cleansing wash” step consisted of running through the steps of the experiment described below, but with a blank inoculate. This technique is used to remove any organisms preexisting on hands and strip the hands of excess dirt and oils, which have been shown in previous studies to create systematic differences between the first and subsequent rounds of testing [54]. This step also gave volunteers an opportunity to practice wetting their hands with the suspension in a way that minimized loss.

**Fig 1:**
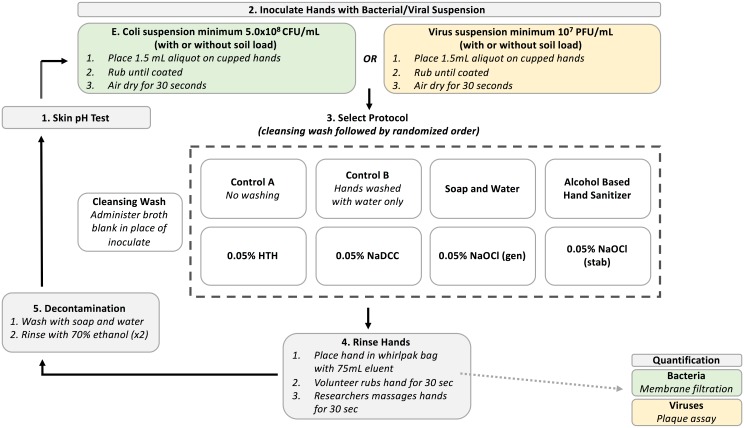
Experimental design.

On each day, testing was performed for all eight handwashing protocols in randomized order according to the same five steps (Fig 1): 1) skin pH testing; 2) spike hands; 3) handwash; 4) hand rinse; and, 5) decontamination.

#### 1. Skin pH testing

The pH of skin on the palm and in the webspace between the pointer and middle fingers on each volunteer’s hand was measured before each round of testing using a Hanna Instruments HI 99181 portable pH meter for skin (Hanna Instruments, Woonsocket, RI).

#### 2. Spike hands

After pH testing, volunteers’ hands were inoculated with a suspension of the test organism. Volunteers were asked to cup their hands carefully, and 750μL of inoculate was slowly pipetted into each palm for a total of 1.5mL. Volunteers then gently rubbed their hands together until all surfaces of hands were coated with the inoculate. Finally, volunteers held hands still and away from the body for an additional 30 seconds to allow the inoculate to dry.

#### 3. Handwashing

**Fig 2:**
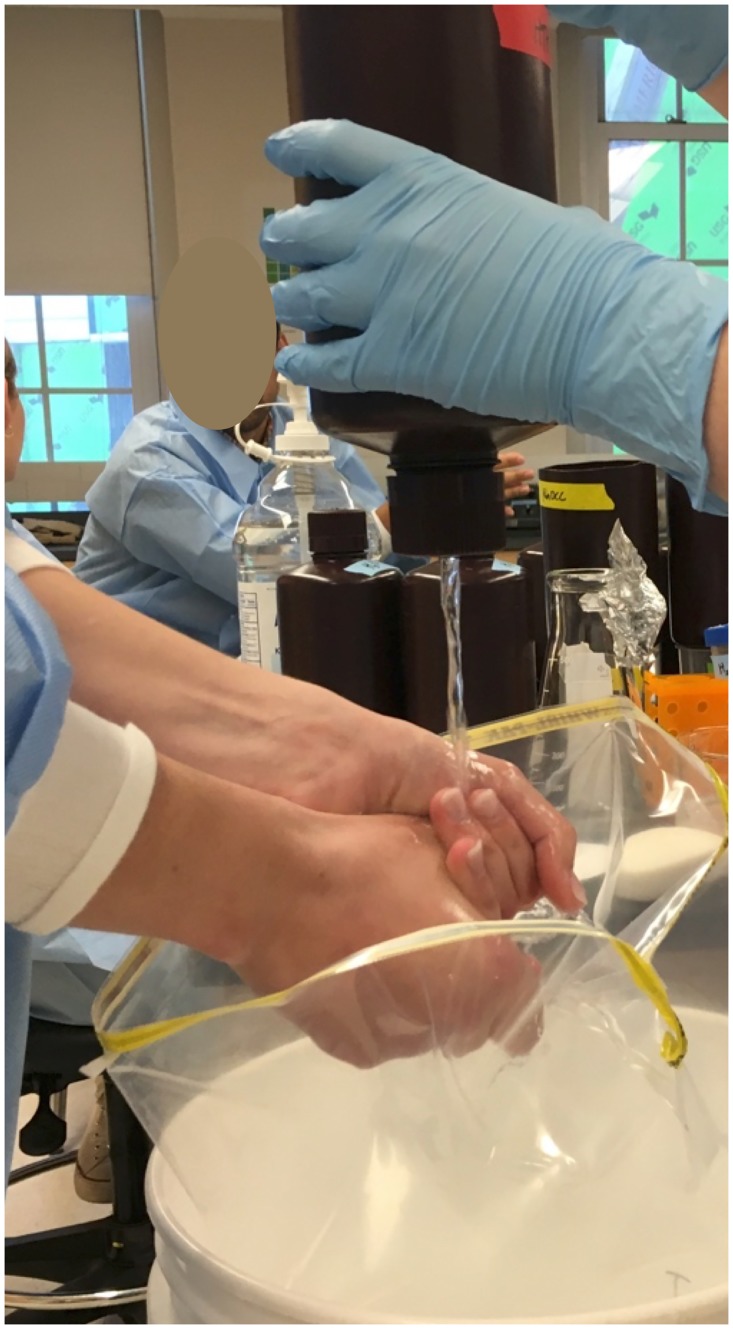
Handwashing station.

The order in which handwashing protocols were performed was randomly assigned for each volunteer prior to testing. Funnels sized to produce a flow rate of 1.5L/minute (the approximate rate of a tap from a bucket commonly used in outbreak settings) were used to administer water for handwashing. A large, 5.4L capacity Whirl-Pak bag (Nasco, Fort Atkinson, WI) was placed in a bucket underneath hands while washing to catch all liquid runoff from the washing process (Fig 2). All handwashing materials and water were held for 24 hours prior to testing to bring them to room temperature (approximately 21°C).

For control A, no handwashing was performed. For control B, hands were washed using water alone; 500mL of water was run through the funnel in 20 seconds while volunteers rubbed their hands under the water taking care to rub all surfaces of hands, in between fingers, under nails, and both the palm and back of hands. For HWWS, volunteers’ hands were wet with 10mL of water and they were provided with a bar of soap and asked to rub the bar in their hands until lathered (about 5 seconds). Volunteers then rubbed their soapy hands together for an additional 20 seconds, taking care to rub all surfaces, and rinsed their hands thoroughly as 500mL of water was run through the funnel in 20 seconds. For ABHS, a quarter-sized volume of sanitizer (average of 2.75g) was placed on the hands and volunteers were asked to rub their hands together until dry, taking care to rub all surfaces of hands. For all chlorine protocols, subjects were asked to rub all surfaces of their hands thoroughly as 200mL of chlorine solution flowed through the funnel (for approximately 8 seconds). To standardize handwashing as much as possible, the WHO instructions for handwashing were demonstrated to each participant prior to handwashing, and during handwashing participants were prompted to ensure that all surfaces of hands were rubbed.

Rinse water was collected during handwashing for the six possible protocols: control, HWWS, and all chlorine solutions. Rinse water collection WhirlPak bags were prepared with 4.5mL of a 12% sodium thiosulfate solution in advance of testing to neutralize any chlorine immediately upon water flowing into the bag. Results from other studies demonstrate that the concentrations of sodium thiosulfate used for sample collection do not have a deleterious effect on bacterial or viral growth [59,60].

#### 4. Hand rinse

After washing was complete and rinse water collected, the hand selected for testing was immediately placed in a smaller WhirlPak bag containing 75mL of eluent up to the wrist. For *E*. *coli*, a 0.1% sodium thiosulfate solution was used as an eluent; for Phi6, PBS with 0.1% sodium thiosulfate was used. Samples were collected using a modified glove-juice method [54,61]: a researcher held the top of the bag tightly around the wrist and volunteers were asked to gently rub their own hand in the solution for 30 seconds, reaching in between fingers and underneath fingernails. A researcher then massaged the bag and hand gently for 30 seconds to ensure that the entire hand was rinsed thoroughly in the eluent. The bag was then sealed and processed within 2 hours.

#### 5. Decontamination

Before repeating the entire process with the next handwashing protocol, volunteers washed their hands thoroughly with soap and water in a sink and dried with paper towels. Volunteers’ hands were then decontaminated with 70% ethanol. Hands were sprayed front and back until coated with ethanol from a spray bottle, and then rubbed and allowed to dry. This was then repeated. Hands were allowed to dry before starting the next round of testing, leading to a wait time of about 10 minutes. During this time volunteers were instructed not to touch anything. Hand rinse samples taken after decontamination during pre-testing confirmed that no organisms remained on hands following this procedure.

### Quantification

Samples were processed using membrane filtration for *E*. *coli* and soft agar plaque assay for Phi6, as described above. Results of zero were replaced with the minimum detection limit of the test as a conservative estimate of the organisms present in the sample.

### Analysis

Laboratory data were collected on paper forms and entered into Microsoft Excel 15 (Redmond, WA, USA). Baseline survey data were collected electronically using Qualtrics software (Qualtrics, Provo, UT) on tablet, mobile phone, and web browser platforms. Difference in the pH of hands prior to testing with each condition was tested used repeated measures ANOVA. The efficacy of the protocols for removal of organisms from hands was defined as the average log reduction in organisms for each handwashing protocol compared to control A (no handwashing), and the log reduction values for each protocol in comparison to no handwashing were then compared to one another. The ability of handwashing protocols to prevent persistence of organisms in rinse water was defined as the average log reduction in organisms compared to control B (handwashing with water only), after which persistence was compared. A one-way repeated measures ANOVA was used to assess the significance of handwashing on log reduction of organisms, and a post-hoc Tukey’s HSD test was used to assess significant differences between each handwashing protocol for models demonstrating significant differences. Bartlett’s test was used to asses each dataset for sphericity, and a Greenhouse-Geisser correction was applied when the test indicated that sphericity was violated. Statistical significance was assessed at the p = 0.05 level. Analysis was performed in STATA 14 (StataCorp LP, College Station, TX).

## Results

Overall, 18 people registered for the study, completed the baseline assessment, and participated in study activities. One volunteer was unable to complete the Phi6 with soil load session due to scheduling conflicts, and the data for six volunteers was lost from the Phi6 without soil load condition due to problems with growth of the bacterial host culture for titration. The final sample sizes for the four conditions were thus 18 for *E*. *coli* without soil load, 18 for *E*. *coli* with soil load, 12 for Phi6 without soil load, and 17 for Phi6 with soil load. Volunteers were 72% female, ranged in age from 18–22 years, and 50% described themselves as “white.”

Across the four testing days, the baseline average pH of hands at the start of the testing day ranged from 4.75–5.06 for palms and 4.79–5.13 for webspaces. The average pH of hands prior to each testing condition ranged from 5.49–6.64 for palms and 5.35–6.50 for webspaces. The pH of hands differed significantly from baseline for 62 of 64 conditions (ANOVA p = 0.009 for webspaces in the *E*. *coli* without soil load treatment, p<0.001 for all other conditions; HTH and NaDCC in webspaces not significantly different at p<0.05 level). There were no significant differences among hands prior to any handwashing treatment (ANOVA p = 0.473 for palms in the *E*. *coli* without soil load treatment, p>0.5 for all others). Overall, pH was higher during Phi6 testing conditions than among *E*. *coli* conditions.

### Hand results

#### *E*. *coli*

**Fig 3:**
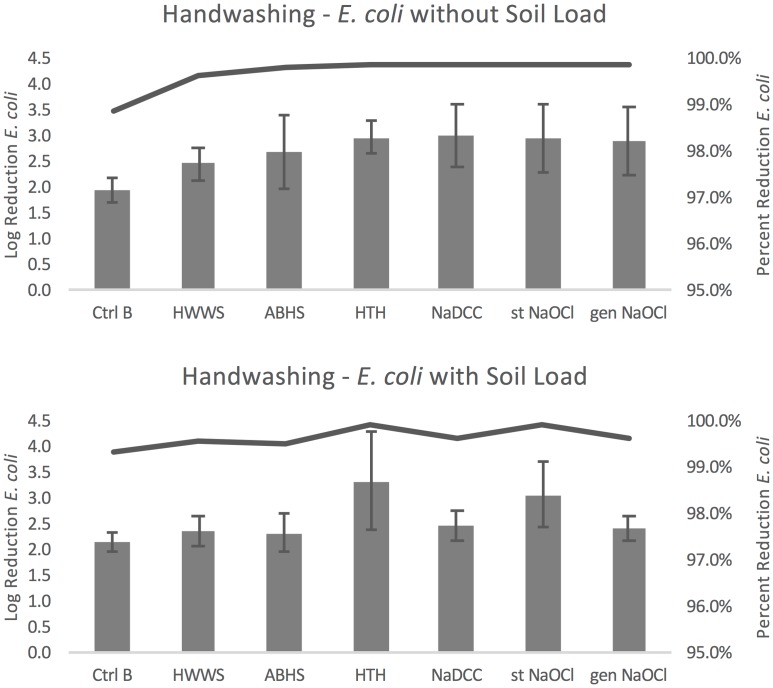
*E*. *coli* handwashing results. Error bars represent standard error of log reduction.

Compared to no handwashing (control A), the seven handwashing protocols resulted in an average log reduction in *E*. *coli* concentration of 1.94–3.01 without soil load and 2.18–3.34 with soil load (Fig 3). For both soil load conditions, handwashing with water only resulted in the smallest reduction (1.94 and 2.18 log). Without soil load, handwashing with NaDCC resulted in the greatest reduction (3.01), and with soil load HTH resulted in the greatest reduction (3.34). Percent reduction in organisms ranged from 98.859–99.902% without soil load and 99.334–99.955% with soil load, as demonstrated by the lines plotted in Fig 3.

Within the seven handwashing protocols there were significant differences in efficacy without soil load (F(6,102) = 2.72, p = 0.034). Handwashing with HTH, NaDCC, and stabilized NaOCl all resulted in greater log reduction than handwashing with water only (p = 0.038, 0.029, and 0.040 respectively). Bartlett’s test indicated that the assumption of sphericity was violated, so the Greenhouse-Geisser correction was used (p = 0.019). There was also a significant difference among handwashing protocols with soil load (F(6,102) = 3.94, p<0.001), with HTH resulting in a significantly greater log reduction than washing with water only, HWWS, and ABHS (p = 0.005, 0.034, and 0.025 respectively).

#### Phi6

**Fig 4:**
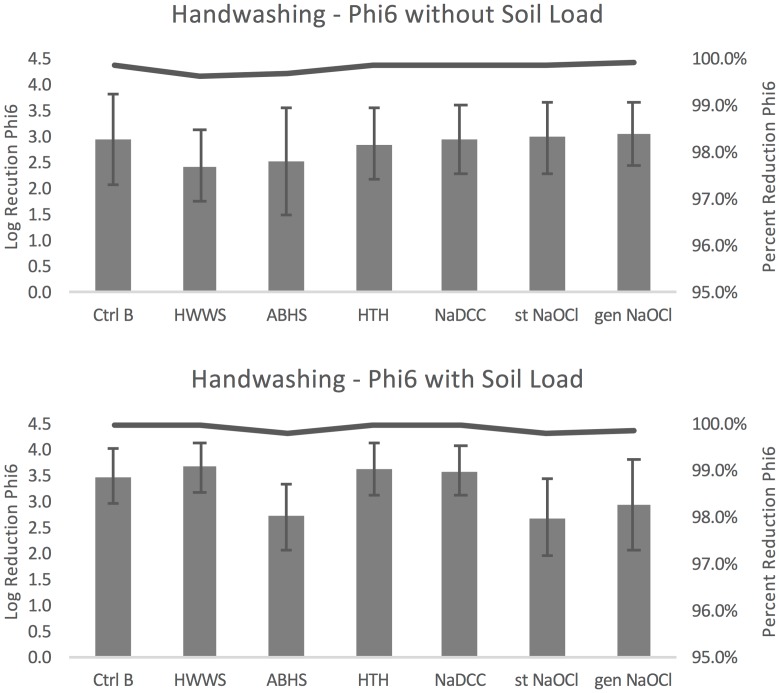
Phi6 handwashing results. Error bars represent standard error of log reduction.

Compared to the condition without any washing (control A), handwashing protocols resulted in an average log reduction in Phi6 concentration of 2.44–3.06 without soil load and 2.71–3.69 with soil load (Fig 4). Without soil load HWWS resulted in the smallest average log reduction (2.44) and generated NaOCl resulted in the largest reduction of Phi6 (3.06). With soil load, handwashing with stabilized NaOCl resulted in the smallest reduction (2.71) and HWWS in the greatest (3.69). Percent reduction in organisms ranged from 99.639–99.913% without soil load and 99.80–99.97% with soil load, as demonstrated by the lines plotted in Fig 4.

Within the seven handwashing protocols there were no significant differences without soil load (F(6,66) = 2.04, p = 0.073). For handwashing with soil load, there were significant differences found (F(6,102) = 7.01, p<0.001). Handwashing with water alone resulted in greater log reduction than ABHS and stabilized NaOCl (p = 0.025 and 0.016, respectively), and handwashing with soap resulted in greater log reduction than ABHS, stabilized NaOCl, and generated NaOCl (p = 0.002, 0.001, and 0.035 respectively). HTH resulted in greater log reduction than ABHS and stabilized NaOCl (p = 0.006 and 0.004, respectively) and NaDCC resulted in greater log reduction than ABHS and stabilized NaOCl (p = 0.006 and 0.004, respectively).

### Rinse water results

#### *E*. *coli*

**Fig 5:**
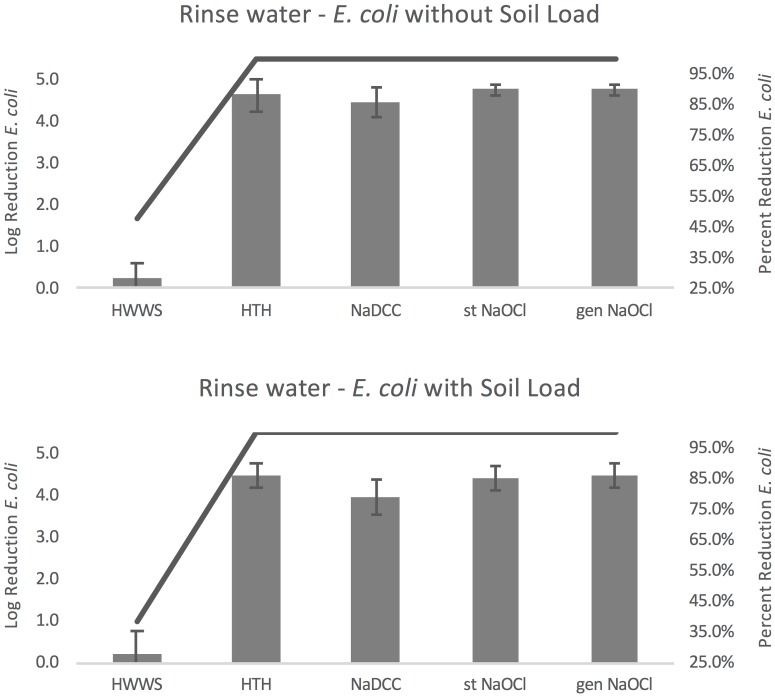
*E*. *coli* rinse water results. Error bars represent standard error of log reduction.

Compared to washing with water only (control B), handwashing resulted in an average log reduction of *E*. *coli* remaining in rinse water of 0.28–4.77 without soil load and 0.21–4.49 with soil load (Fig 5). For both conditions with and without soil load, HWWS resulted in the smallest reduction (0.28 and 0.21). The greatest log reductions were found with stabilized and generated NaOCl for conditions without soil load (both 4.77) and with HTH and generated NaOCl for conditions with soil load (both 4.49). Percent reduction in organisms ranged from 47.691–99.998% without soil load and 38.968–99.997% with soil load, as demonstrated by the lines plotted in Fig 5.

Within the five handwashing protocols there were significant differences with and without soil load (without soil load F(4,68) = 331.7, p<0.001 and with soil load F(4,68) = 162.44, p<0.001). Bartlett’s test indicated that the assumption of sphericity was violated in both models, so the Greenhouse-Geisser correction was used (both p<0.001). In both models all four chlorine solutions resulting in significantly greater log reductions in *E*. *coli* than HWWS (all p<0.001).

#### Phi6

**Fig 6:**
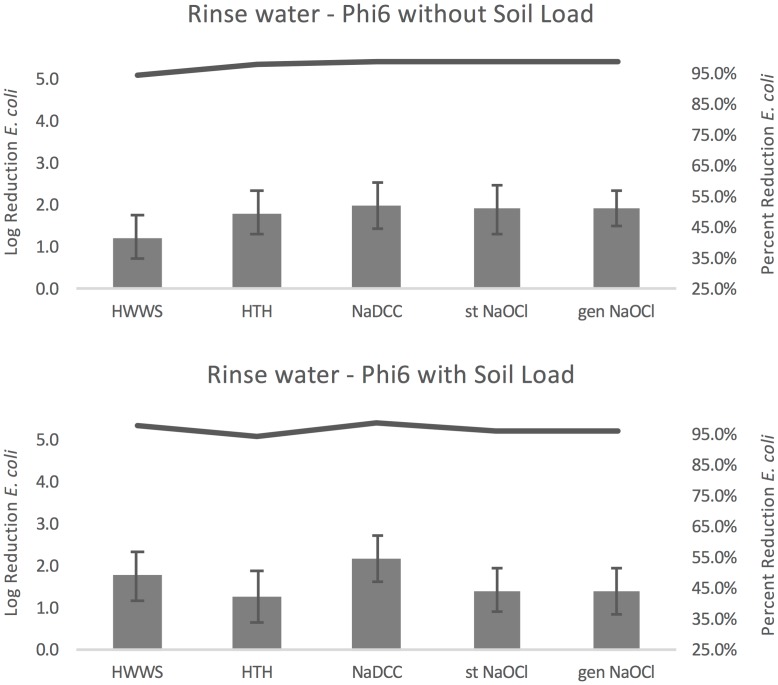
Phi6 rinse water results. Error bars represent standard error of log reduction.

Compared to washing with water only (control B), other handwashing protocols resulted in an average log reduction in Phi6 remaining in rinse water from 1.26–2.02 without soil load and from 1.30–2.20 with soil load (Fig 6). HWWS resulted in the smallest reduction without soil load (1.26) and HTH resulted in the smallest reduction with soil load (2.02). Both with and without soil load NaDCC resulted in the largest reductions in Phi6 concentration (2.02 and 2.20). Percent reduction in organisms ranged from 94.442–99.044% without soil load and 94.938–99.368% with soil load, as demonstrated by the lines plotted in Fig 6.

Within the six handwashing protocols there were significant differences without soil load (F(4,43) = 8.95, P<0.001), and all chlorine solutions resulted in significantly greater log reduction in Phi6 than HWWS (HTH p = 0.002, NaDCC, stabilized and generated NaOCl all p<0.001). There were no significant differences in the log reduction of Phi6 remaining in wastewater with soil load (F(4,67) = 3.35, p = 0.071). Bartlett’s test indicated that the assumption of sphericity was violated in the model with soil load, so the Greenhouse-Geisser correction was used (p = 0.027).

## Discussion

We completed a laboratory-based study in which 18 subjects had their hands contaminated with two surrogate organisms with and without soil load and washed their hands with eight different protocols to: 1) quantify the removal of surrogate organisms from their hands and, 2) quantify the persistence of these organisms in the environment and in rinse water. Across our study, we noted some significant differences between the protocols tested. HTH performed better most consistently across conditions, demonstrating greater log reduction than at least one other method in all three significant handwashing models. HWWS performed well for Phi6 with soil load, outperforming three other conditions. However, these handwashing efficacy differences were small in magnitude. These results demonstrate that concerns that chlorine in particular would not be efficacious due to chlorine demand from skin seem unlikely, though there were small efficacy differences between particular chlorine solutions. Significant differences found for persistence of organisms in rinse water were more pronounced than those for handwashing, and the larger magnitude of differences in log reduction of organisms in rinse water demonstrates that chlorine use may have an added benefit of preventing ongoing disease transmission through rinse water.

Despite these statistical differences, we would caution against over interpretation of the data, as confidence intervals for the significant differences in handwashing tended to be large, ranging from < 0.5 log to > 1.5 log reduction. We suspect that the rough similarity in efficacy among protocols is because much of the removal and inactivation from handwashing is due to mechanical action; some studies have shown that the addition of an agent such as soap to handwashing with water alone only marginally improves efficacy [39,40]. Thus, our results are consistent with the limited prior research and provide evidence that—contrary to international agency concerns—none of the six handwashing protocols are consistently more or less efficacious than others, and all can be recommended to reduce transmission of infectious disease. We recommend responders select the handwashing method most acceptable and appropriate for their context, while considering the possible differences among types of chlorine solutions and the benefit of chlorine in preventing ongoing transmission via rinse water.

To our knowledge, this is the first study investigating the persistence of organisms in handwashing rinse water. Rinse water may be less important when water is carried by a drainpipe to a treatment facility or when diseases are less transmissible and virulent. However, it may be an important concern for diseases with outbreak potential such as Ebola and cholera, especially in low-resources or emergency contexts. While there has not been any documented environmental spread of Ebola, the infectious dose is very low and the origin of some cases remains unexplained [5,62,63]. We observed a dramatic reduction in *E*. *coli* when chlorine was used for handwashing (mean log reduction > 4 log) but very little reduction with HWWS, and a less dramatic but statistically significant reduction in Phi6 (without soil load, average approximately 0.6 log reduction). For Phi6 with soil load, log reduction was similar across all conditions.

The striking difference in significant results and the magnitude of these rinse water results that we observed between *E*. *coli* and Phi6 is likely due to differences in mechanisms of disinfection acting on bacteria and viruses. Chlorine inactivates bacteria by lysing the cell [64], and it probably inactivates viruses by reacting with viral proteins, and, to a lesser extent, genomic material [65,66]. Phi6 and similar viruses such as Ebola are more fragile than bacteria and may be inactivated by soaps and surfactants that disrupt the lipid membrane and capsid proteins [67,68], and enveloped viruses have been demonstrated to be especially volatile and subject to decay on human skin [69,70]. However there is still significant uncertainty about the mechanisms of inactivation, given that effects can vary greatly even among similar viruses [71]. We suspect that the reduction in Phi6 with HWWS was greater than the reduction in *E*. *coli* because the bacteriophage may be more susceptible to the mechanical action of handwashing, interaction with skin, and lipophilic action of soap, but that the reduction was less in chlorine because Phi6 was more resistant to chlorine than *E*. *coli* on hands. Because the chlorine was neutralized immediately in collection bags pre-dosed with sodium thiosulfate our results present a very conservative estimate of the ability of chlorine to limit persistence in rinse water, and it is possible this could be increased by longer contact with the chlorine. Therefore, the use of chlorine for handwashing may have an added benefit of inactivating organisms removed from hands and preventing persistence in the environment.

Our observations on hand pH were consistent with previous research, showing a transient increase in the pH of hands after handwashing. Overall, pH of hands was significantly higher after handwashing than at baseline, as expected due to the stripping of sebum and oils that occurs during the washing process [72,73]. pH also rose more during Phi6 conditions than it did during *E*. *coli* testing. There were no differences among pH of hands prior to any of the handwashing conditions, meaning that the cleansing wash served the purpose of eliminating this potentially confounding difference in pH between handwashing conditions.

Our study is limited by differences in the characteristics of the laboratory at Tufts University and hospitals and communities in EVD outbreaks. We conducted the study in rooms maintained at a temperature around 21°C, which is lower than typical conditions in EVD affected areas that experience high humidity and temperatures above 30°C. The temperature of the water used for handwashing, also kept at room temperature in our study, would likely be warmer in a more tropical climate. Previous research has shown that the use of warm or cold water for handwashing has no significant impact on efficacy, given that water hot enough to destroy bacteria would also painfully harm skin [27,74]. Additionally, our subjects were college students, and it is possible that age or occupation could impact the texture of skin or retention of microorganisms. Unfortunately, we were also limited by the smaller sample size for Phi6 without soil load, due to loss of samples.

There also may be differences in handwashing in the laboratory and in the field. While we controlled handwashing conditions to the best of our ability and instructed and observed volunteers carefully, handwashing cannot be replicated exactly from person to person. Furthermore, the efficacy of these protocols in the field would likely be lower as proper handwashing technique might not be adhered to as stringently. We also did not examine different drying methods, although it has been shown that different approaches to drying can reduce contamination further or potentially re-contaminate hands [75].

Finally, the greatest limitation of our study was the need to use surrogates rather than the Ebola virus for the safety of participants; this limitation cannot be ethically overcome, due to the danger the Ebola virus poses to humans. The mechanisms of disinfection and removal of viruses from hands are not clear [65,71], so while we have chosen a surrogate that we believe is most likely to mimic the dynamics of the Ebola virus for handwashing our conclusions are limited by practicality and safety. We believe that the use of this surrogate provides the best possible information on handwashing, particularly as there is work demonstrating that in vitro testing on surrogate surfaces such as pig skin (on which the actual Ebola virus could be tested) produce results that do not match those found on human hands [69]. Nonetheless, surrogate surfaces may provide a valuable point of comparison, and further illumination of the mechanisms of viral inactivation for Ebola can guide and validate selection of surrogate organisms.

While our study provides valuable insight about handwashing for both viral hemorrhagic diseases such as Ebola and common bacterial organisms, there are many fruitful areas for future research. We assessed six of handwashing protocols most commonly used during the EVD outbreak in West Africa, but there are many other ways of handwashing that could be investigated. These include (but are not limited to) handwashing with differing soap formulas, iodine-based scrubs, alcohol-based foams and wipes, traditional practices such as using ash or sand, and handwashing of gloved hands with any of these approaches. We observed many significant differences in the condition with Phi6 and soil load in particular—as these differences don’t represent one clear pattern and tend to be low magnitude, further research is needed to understand the source and relevance of these differences. For example, the currently recommended handwashing protocols result in different lengths of handwashing among methods, and in the field compliance issues often mean handwashing is not completed for the full recommended time. Further research is also needed to assess critical thresholds for the length of time these handwashing protocols are performed, as handwashing time may have an impact on efficacy. Finally, further research should include a broad range of organisms to add to the small body of literature about handwashing efficacy for different pathogens of concern.

Our results suggest all handwashing protocols tested are likely to be similarly efficacious for handwashing in EVD contexts, and that concerns that chlorine in inefficacious for handwashing do not have a strong basis in evidence. HTH may be considered as a particularly good choice for response in which use is acceptable and logistically feasible, as it consistently performed well against both organisms of interest. Overall, chlorine may be preferable in some cases because it may confer a benefit of continued disinfection in rinse water after handwashing. In related work, we investigated the safety of these handwashing methods based on their potential to irritate skin and pose a risk for disease transmission, and found no clinically-relevant differences between these same six methods [76]. Based on our results that validate both the safety and efficacy of all six commonly used handwashing protocols, we recommend Ebola responders and communities facing outbreaks focus on what is most practical and choose the most acceptable and sustainable methods to allow for consistent and thorough handwashing.

## Supporting information

S1 Dataset *E*. *coli* data. (XLS)

S2 Dataset Phi6 data. (XLS)
